# Supplementary material for: IGF-1 regulates cancer cell immune evasion in prostate cancer
Source: Sci Rep. 2025 Nov 3;15:38422. doi: 10.1038/s41598-025-22288-5 (PMC12583730; doi:10.1038/s41598-025-22288-5)
Supplement: Supplementary file 2 — Supplementary Material 2 [file 41598_2025_22288_MOESM2_ESM.pdf]

Nandakumar et al Supplementary Figures

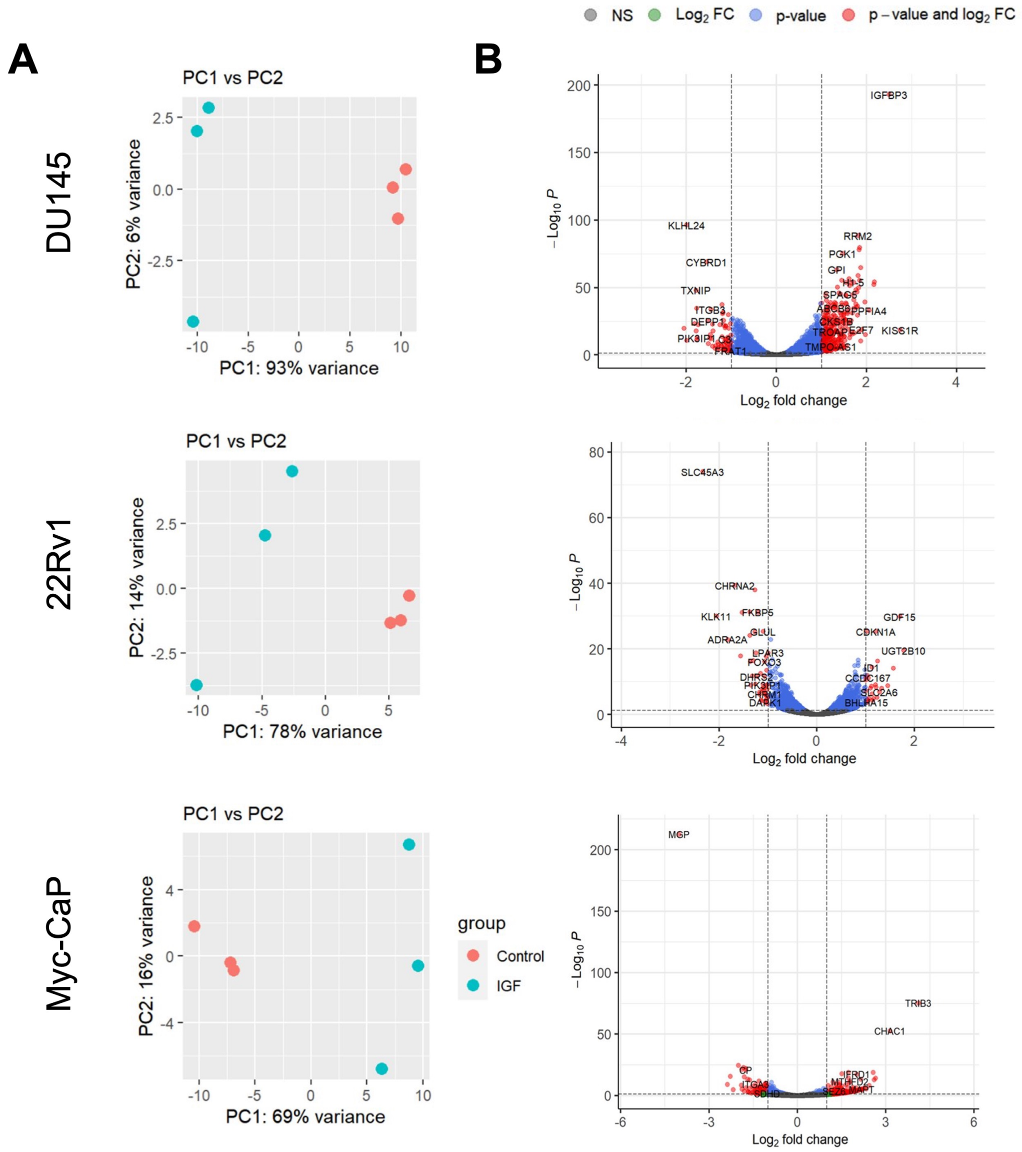

Supplementary Figure S1. Effect of IGF-1 on transcriptome of PCa cells. DU145, 22Rv1 and Myc-CaP cells were serum starved for 24 hrs, treated with 30 nM IGF-1 or solvent (control) for 24 hrs and RNA was extracted for sequencing. **A.** Principal component analysis (PCA) of RNA sequencing data (top 500 genes) for the three cell lines displaying the variation of each sample with respect to their transcriptomic profile. The x-axis shows the first principal component (PC1), accounting for the largest amount of variation in the experiment and the y-axis shows the second principal component (PC2). **B.** Volcano plots displaying the top high and low differentially expressed genes.

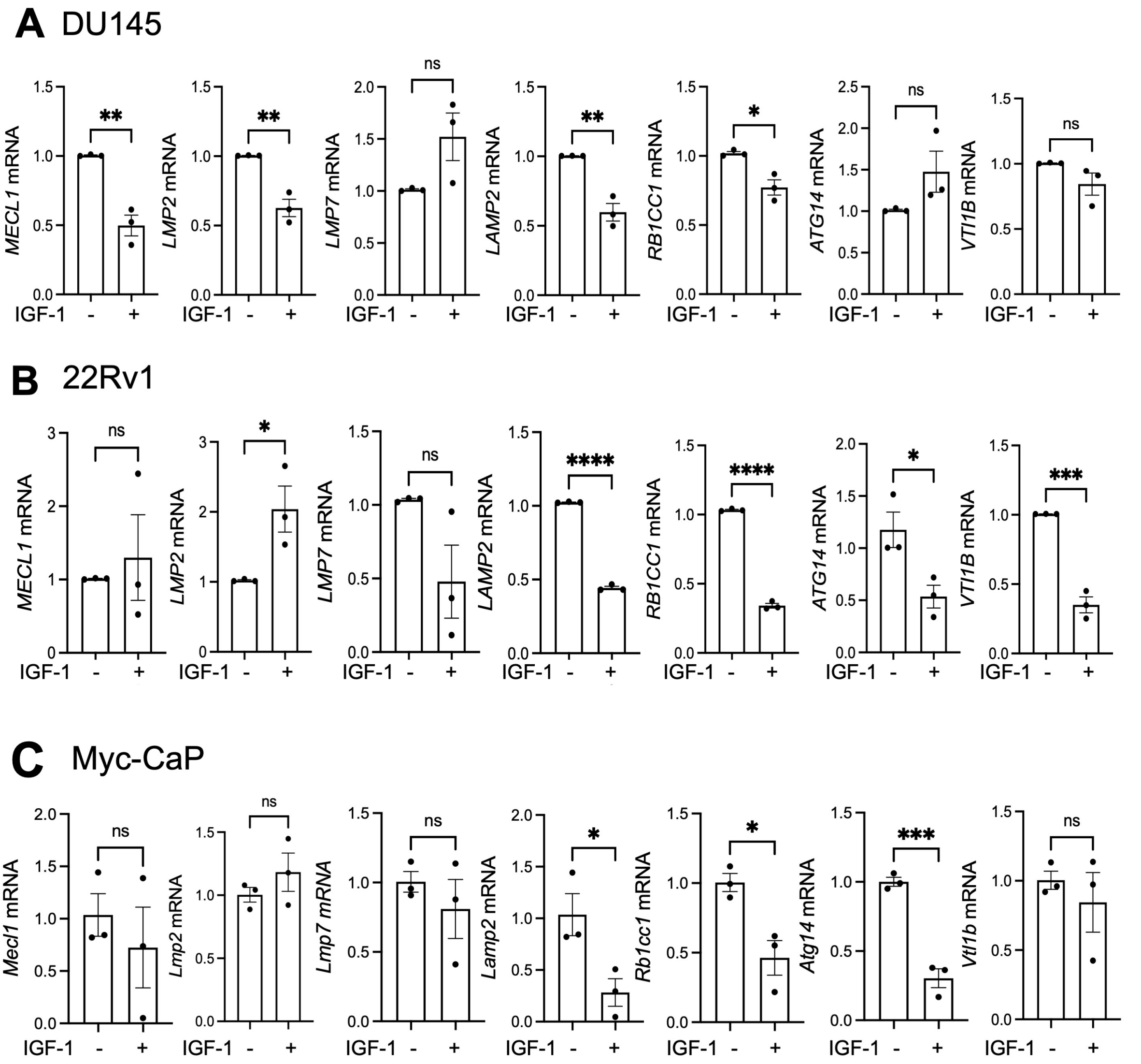

Supplementary Figure S2. IGF-deregulated genes that influence protein degradation. PCa cells **A.** DU145, **B.** 22Rv1 and **C.** Myc-CaP were treated for 24 hr with 30 nM IGF-1 or solvent (control) and tested for expression of the indicated genes by RT-qPCR. Graphs: mean ± SEM of three independent analyses. *p<0.05; **p<0.01; ***p<0.001; ****p<0.0001; ns, nonsignificant.

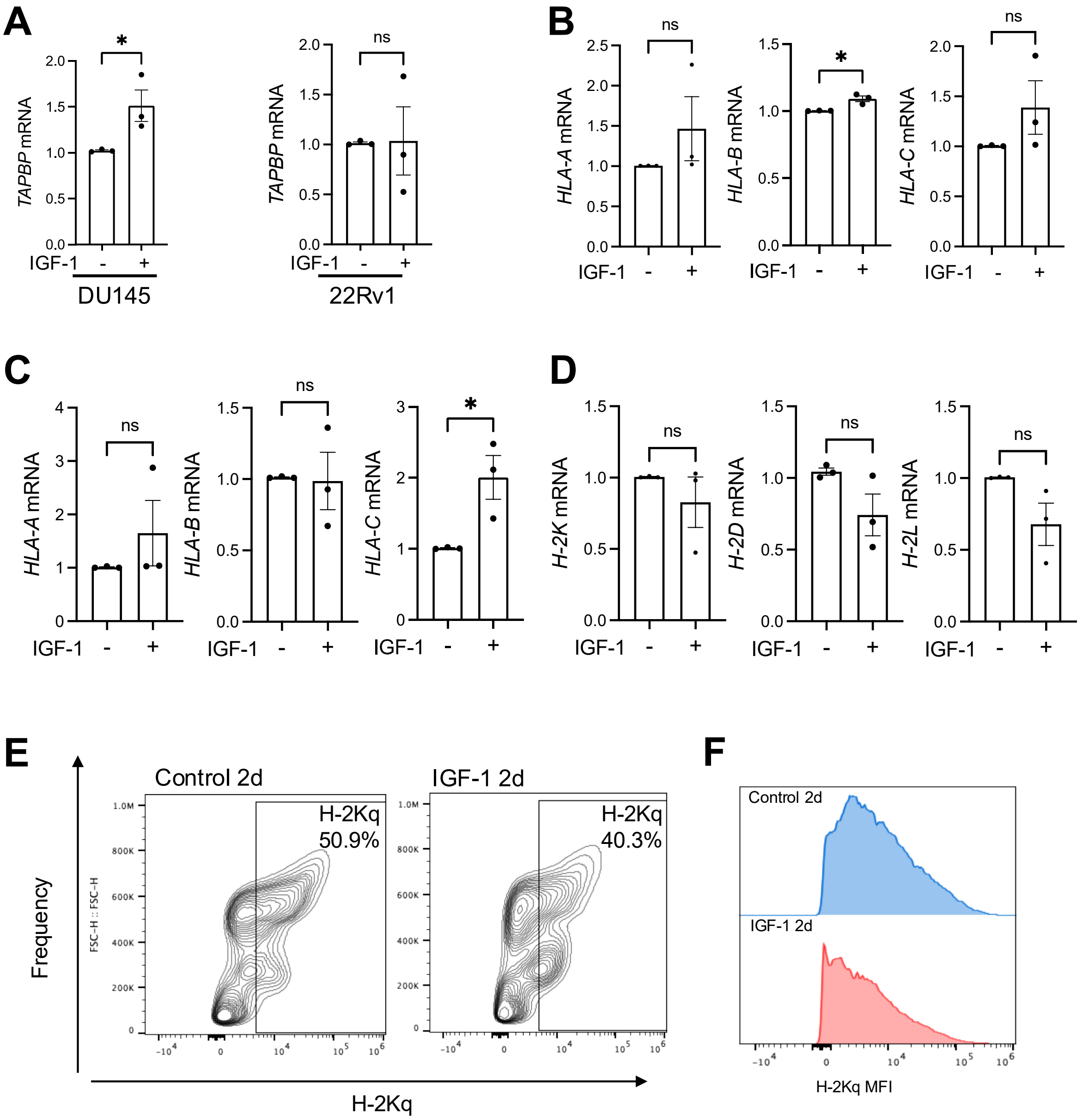

Supplementary Figure S3. IGF-1 does not cause consistent deregulation of Class I genes in human or murine PCa cells. PCa cells were serum starved for 24 hrs and treated with solvent (control) or 30 nM IGF-1. **A,** Effects of IGF-1 on *TAPBP* mRNA expression on left, DU145; right, 22Rv1. **B-D**. Effects of IGF-1 on expression of Class I alleles in: B, DU145; C, 22Rv1; D, Myc-CaP. Graphs represent mean ± SEM of three independent analyses (*p<0.05; ns, nonsignificant). **E-F**. Representative flow cytometry plots of H2Kq percentage positivity (E) and MFI surface expression (F) in Myc-CaP cells treated with 30 nM IGF-1 or solvent after 2 days.

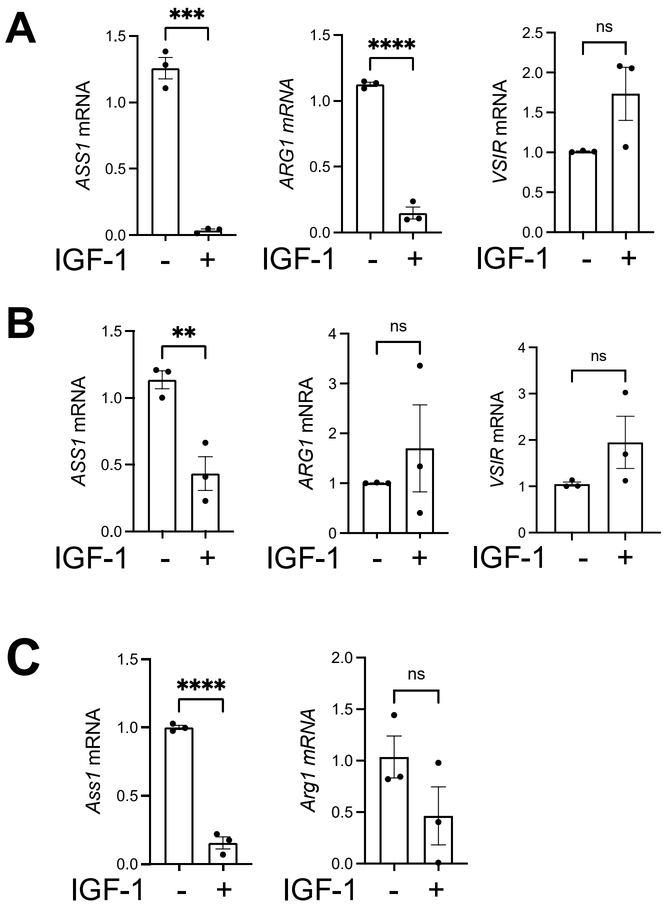

Supplementary Figure S4. Effects of IGF-1 on expression of immune suppressors. Prostate cancer cells were serum starved for 24 hrs and treated with solvent (control) or 30 nM IGF-1. **A-C.** Effects of IGF-1 on *ASS1*, *ARG1* and *VSIR* expression assessed by qRT-PCR in DU145 (A), 22Rv1 (B) and Myc-CaP (C). Vsir mRNA expression was undetectable in Myc-CaP. Graphs represent mean ± SEM (n=3 independent samples.*p< 0.05; **p<0.01; ***, p<0.001; ****, p<0.0001; ns, nonsignificant.

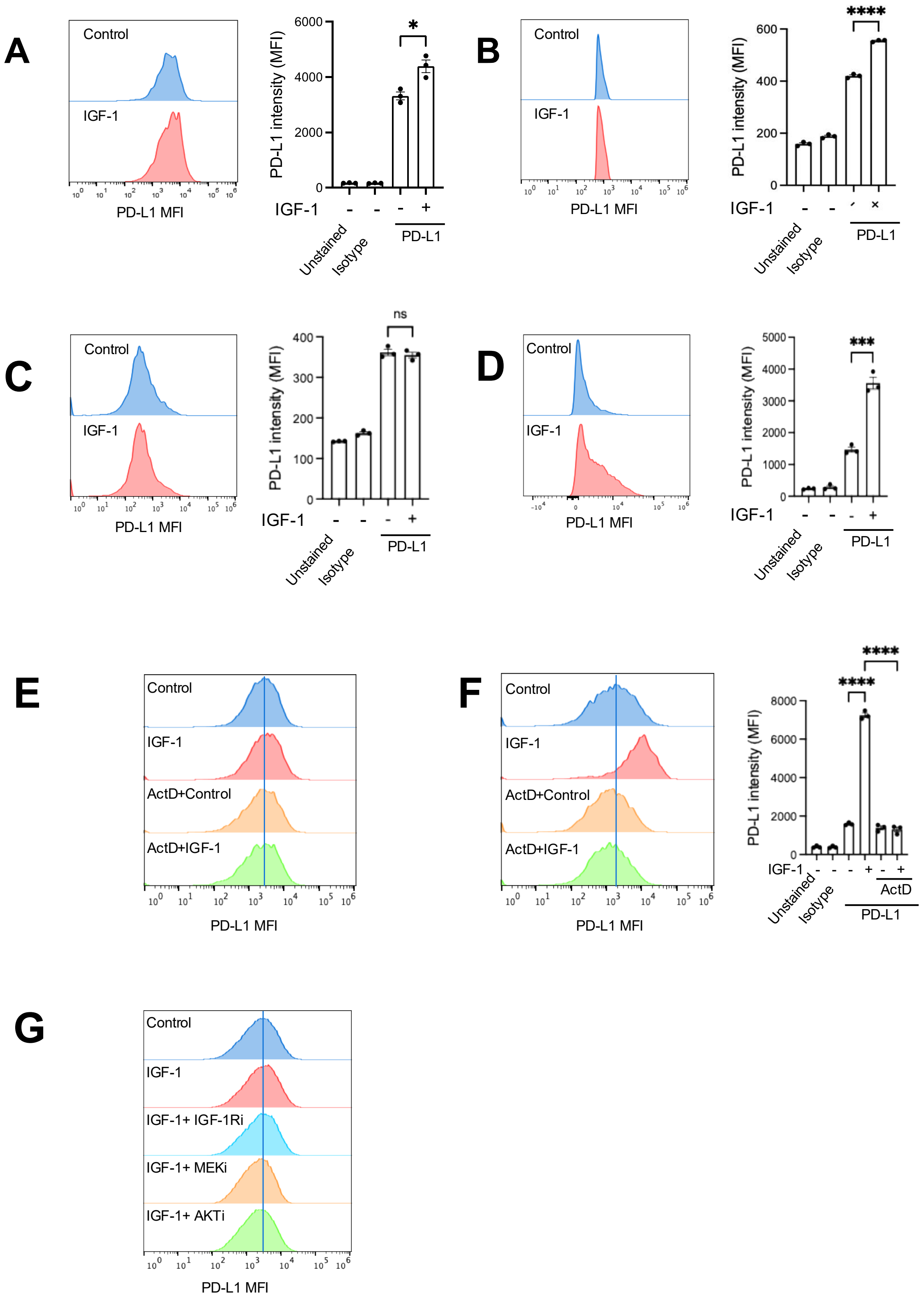

Supplementary Figure S5. Effects of IGF-1 on PD-L1 surface expression. A. DU145 cells were treated with 30 nM IGF-1 or solvent (control) in full medium (10% FBS) and PD-L1 surface expression measured using flow cytometry. **B-D.** Cancer cells were serum starved for 24 hrs and treated with solvent (control) or 30 nM IGF-1. Effects of IGF-1 on PD-L1 surface expression was measured using flow cytometry on prostate cancer cell lines Myc-CaP (B) and 22Rv1 (C), and colorectal cancer cell line H747 (D). Left, representative histogram; right, expression

measured as MFI, mean ± SEM from 3 independent experiments. **E.** Flow cytometry histogram of PD-L1 surface expression in DU145 cells pre-treated with 1 µg/mL actinomycin D for 2 hours, followed by the addition of 30 nM IGF-1. PD-L1 expression was assessed 24 hours later. Blue vertical line indicates PD-L1 MFI in untreated control cells **F.** H747 cells were treated as in E. and PD-L1 surface expression measured. Left, representative histogram with blue vertical line indicating PD-L1 MFI in untreated control cells; right, expression measured as MFI, mean ± SEM from 3 independent experiments. **G.** Flow cytometry histogram of PD-L1 surface expression in DU145 cells pre-treated for 1 hour with 300 nM IGF-1R inhibitor BMS-754807, 5 µM AKT inhibitor AZD5363, or 50 nM MEK inhibitor trametinib, followed by 30 nM IGF-1 stimulation for 24 hours. Blue vertical line indicates PD-L1 MFI in untreated control cells. Graphs represent mean ± SEM (n=3 independent samples.*p< 0.05; **p<0.01; ***, p<0.001; ****, p<0.0001; ns, nonsignificant).

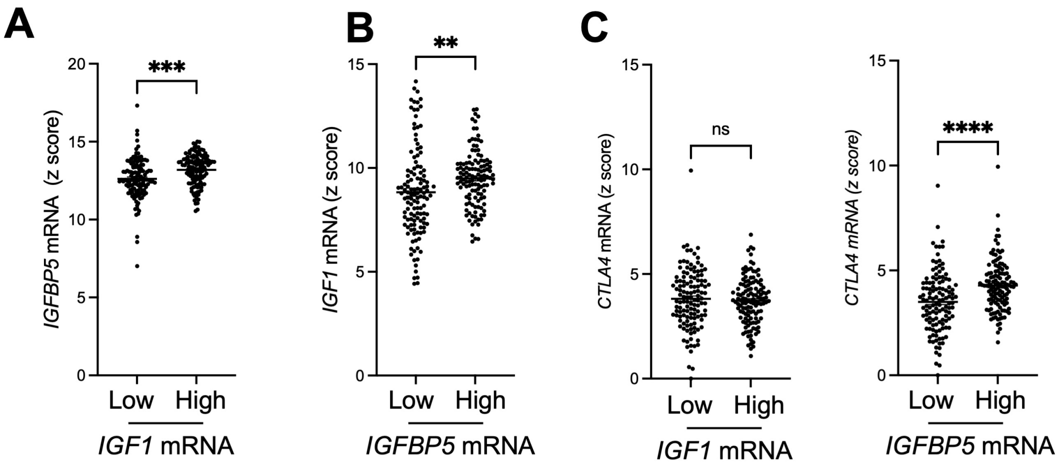

Supplementary Figure S6. Association of IGF axis activation markers with immune checkpoints in clinical prostate cancers. A, B and C, Tumours from TCGA prostate adenocarcinoma (n=498) were divided based on highest and lowest quartiles of tumour endogenous *IGF1* (n= low:124, high=125) and *IGFBP5* (n= low: 124, high =125) mRNA (Z score relative to all samples). A, Association between *IGF1* mRNA and *IGFBP5* mRNA in prostate cancer tissues. B, Association between *IGFBP5* mRNA and *IGF1* mRNA in prostate cancer tissues. C, Association between *IGF1* (left) and *IGFBP5* (right) mRNA and *CTLA4* mRNA expression in prostate cancer tissue. *p<0.05; **p<0.01; ***p<0.001; ****p<0.0001; n.s., nonsignificant.

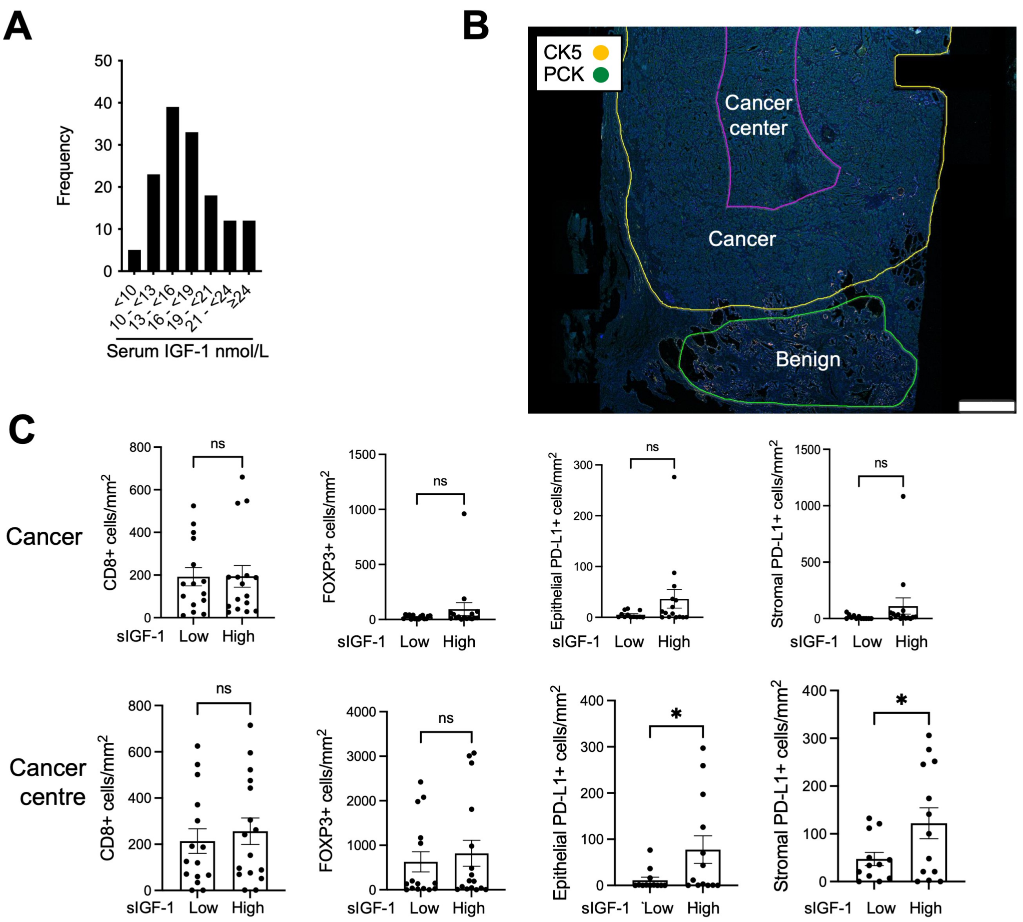

Supplementary Figure S7. IGF axis activation associates with immune checkpoints and infiltrates in clinical PCa. A. Serum IGF in 139 men with localized PCa recruited to ProMPT study. Assay used IDS-iSYS IGF-I assay (Immunodiagnosticsystems). All values were within normal range (7.0-31.7 nmol/L) for males >32 yr [1]. **B.** PCK and CK5 from mIF of radical prostatectomy, marked up for benign, cancer, cancer center. **C.** Quantification of CD8+ T-cells, FOXP3+ Tregs, epithelial and stromal PD-L1+ cells in cancer (upper) and cancer centre (lower) in high vs low serum IGF-1 (sIGF-1) patients. Numbers in FOXP3+, CD8+ analysis: n= 15 low, 16 high sIGF-1 (1 lost due to technical issues); PD-L1+: n= 12 low, 13 high sIGF-1 patients (7 lost).

Supplementary references

1. Bidlingmaier, M. *et al.* Reference intervals for insulin-like growth factor-1 (igf-i) from birth to senescence: results from a multicenter study using a new automated chemiluminescence IGF-I immunoassay conforming to recent international recommendations. *J Clin Endocrinol Metab* **99**, 1712-1721 (2014). https://doi.org/10.1210/jc.2013-3059
